# Effectiveness of School-Based Interventions in Europe for Promoting Healthy Lifestyle Behaviors in Children

**DOI:** 10.3390/children10101676

**Published:** 2023-10-11

**Authors:** Archontoula Drouka, Dora Brikou, Céline Causeret, Nur Al Ali Al Malla, Stéphane Sibalo, Concha Ávila, Gabriela Alcat, Anastasia E. Kapetanakou, Patricia Gurviez, Nawel Fellah-Dehiri, Marine Masson, Meropi D. Kontogianni, Mary Yannakoulia

**Affiliations:** 1 Department of Nutrition & Dietetics, Harokopio University, 17671 Athens, Greece; drouka.ar@gmail.com (A.D.); dwramprikou@gmail.com (D.B.); mkont@hua.gr (M.D.K.); 2 VIF (Vivons en Forme), 59350 Lille, France; celine.causeret@vivonsenforme.org (C.C.); stephane.sibalo@vivonsenforme.org (S.S.); 3 Comocomoschool, 28049 Madrid, Spain; nur@comocomoschool.com; 4 Federación Española de Industrias de Alimentación y Bebidas, 28001 Madrid, Spain; c.avila@fiab.es (C.Á.); g.alcat@fiab.es (G.A.); 5 Federation of Hellenic Food Industries, 15451 Neo Psychico, Greece; natkap@sevt.gr; 6 Agroparistech, INRAE, Paris-Saclay University, 91120 Palaiseau, France; patricia.gurviez@agroparistech.fr (P.G.); nawel.fellah-dehiri@agroparistech.fr (N.F.-D.); marine.masson@agroparistech.fr (M.M.)

**Keywords:** primary school, nutrition, physical activity, children, obesity

## Abstract

The objective of this narrative review was to summarize existing literature on the effectiveness of school-based interventions, implemented in Europe, under the aim of promoting healthy lifestyle behaviors in children (6–10 years old). A search of PubMed, Scopus, EFSA and Google Scholar databases was performed for studies published from January 2016 to June 2022. Specific search terms and exclusion criteria were used. Based on the results, diet and physical activity interventions had favorable effects on a series of health outcomes, including anthropometric parameters, biomarkers, eating behavior and self-efficacy. Diet-only interventions had a positive impact specifically on eating habits, mostly on water consumption. Most successful interventions lasted for 1 school year, and they were characterized by parental involvement and teachers’ training.

## 1. Introduction

Childhood is one of the critical periods for good health and development in human life. During childhood, physiological needs for nutrients increase and the adoption and maintenance of high-quality eating habits is particularly important [1]. A healthful diet during childhood promotes growth and cognitive development of children and may contribute to the prevention of chronic diseases in later life [2,3]. Similarly, regular physical activity is associated with physiological and mental health benefits, including a low risk of adiposity, improved fitness and optimal cardiometabolic health [4,5]. Evidence suggests that both eating and physical activity habits adopted early in life track to some extent into adulthood [6,7]. It is therefore important to establish healthy lifestyle behaviors as early as possible during lifetime. However, dietary consumption surveys show that most children in Europe do not meet these guidelines [8,9] and a great proportion of children spend less than the recommended 60 min of moderate-to-vigorous physical activity per day [10].

Recent figures also show alarming and increasing numbers of children with overweight or obesity in Europe. Childhood obesity is associated with several short-term physical health consequences, such as adverse blood lipid profile, altered glucose metabolism and obstructive sleep apnea, as well as long-term effects, i.e., higher risk for hypertension, diabetes mellitus, cardiovascular disease, gallbladder disease and osteoarthritis in adulthood [11,12,13,14]. The presence of overweight and obesity during childhood has also been linked with psychosocial adverse consequences, including poor self-image, low self-esteem, higher risk for eating disorders and poor quality of life [15]. The increasing prevalence of childhood obesity can be linked to social and lifestyle changes in Europe occurring over the last three decades with the development of unhealthy eating habits (characterized by a high intake of unhealthy lipids and added sugars, as well as by low consumption of complex carbohydrates and fiber) and sedentary way of living (high screen time and low engagement in lifestyle physical activities) [11]. To date, many programs have been developed to promote healthy lifestyle and prevent obesity in children. The vast majority of these programs use schools as the optimal setting for the implementation of interventions targeting school-aged children [16].

Schools are a crucial social environment for children and many attempts have been made to utilize this environment to promote healthful behaviors in youth, including eating and physical activity habits [17,18,19]. School-based interventions have the potential to reach almost 100% of children of diverse ethnic and socio-economic groups. Other influencing factors of eating behaviors in the school environment are the availability of food and beverages, apart from meals served in many countries (e.g., foods provided in vending machines and school stores), and the provision of nutrition education classes. Additionally, schools can promote physical activity as structured physical activity, while sports education is a mandatory part of the school curriculum, and, in many cases, children have to walk from and to school on week days [20]. Thus, schools represent an ideal setting to promote and provide both healthy nutrition and physical activity education [17,21].

Previous reviews of school-based interventions have demonstrated a variety of intervention approaches, including nutrition knowledge classes, changes in the availability of healthy foods in school canteens, food programs providing free foods, environmental changes, parental involvement and teachers’ training, delivery modes and intensities, and outcome measurements. Additionally, no recent (in the last 10 years) review has been published so far that includes the latest interventions implemented in the European continent, given that the socio-economic context and school systems are different in most European countries compared to the U.S. or other countries in the American continent or in Asia. This is also true for physical activity and the eating environment. Therefore, reviews focusing on school interventions that share common geoenviromental and cultural background are necessary, as already pointed out in past reviews published in early 2010s [21,22]. Thus, the aim of the present narrative review is to evaluate the most recently published school-based interventions for promoting healthy lifestyle behaviors in Europe and potentially identify common components in the successful ones in comparison with those that did not produce the expected results in lifestyle behaviors.

## 2. Methods

### Strategy Used

A search of PubMed, Scopus, EFSA and Google Scholar databases was performed for studies published from January 2016 to June 2022. The search terms were ‘school based intervention’, ‘diet’, ‘nutrition’, ‘nutritional program’, ‘food education’, ‘hydration’, ‘water consumption’, ‘physical activity’ and ‘exercise’ used individually and in combination (‘school based interventions and diet or nutrition program or food education’, ‘school based interventions and physical activity’, ‘school based interventions and water consumption or hydration’).

Studies were included if they (i) investigated the effectiveness of a school-based intervention targeting PA behavior and/or nutrition behavior (NB); (ii) were clinical trials; (iii) targeted children aged 6 to 10 years; (iv) were conducted in Europe; and (v) were written in the English language.

Observational studies or reviews and family-based interventions were excluded.

## 3. Results

### 3.1. Search Outcomes

**Figure 1:**
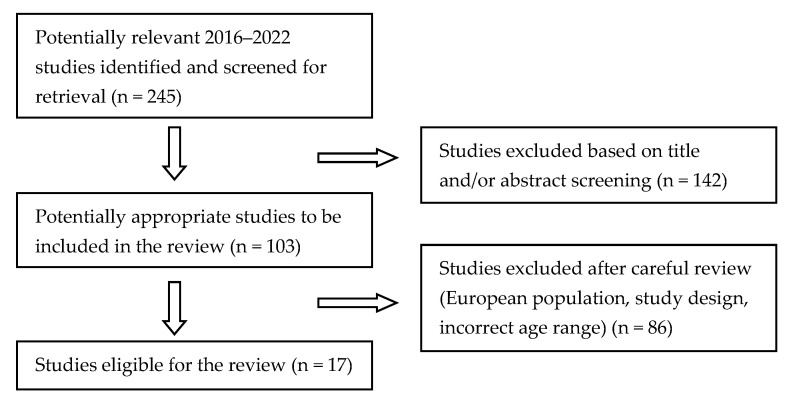
Flow diagram: school-based interventions.

Figure 1 summarizes the review article selection. The initial database search yielded 245 publications. After screening titles and/or abstracts, the total number was reduced to 103. After a careful review, 86 publications were excluded because they did not meet one or more of the inclusion criteria. Almost all of the excluded publications were studies that were not conducted in Europe or targeted preschool children or adolescents.

The seventeen studies included in the current analysis are presented in Table 1 and Table 2, including their target population and their design, duration and type of intervention (diet only or diet and physical activity). The interventions in the tables are presented based on their number of participants in a descending order. Publications referring to the same project were grouped together. These interventions’ duration ranged from 5 weeks to 2 years. Of the 17 included studies, 8 examined interventions involving diet and physical activity and the other 9 examined diet only.

An intervention was considered to be successful if it had an impact on any kind of health outcome (anthropometric measurements, biomarker, dietary and physical activity habits). Interventions resulting in food knowledge improvement were not categorized as successful. Therefore, of the included 17 studies, 11 were defined as successful and 6 as unsuccessful.

### 3.2. Diet-Only Interventions

Most of the successful diet-only interventions examined the potential association of a series of school-based interventions that included and targeted only water intake (two studies) [23,24] or water intake and nutrition education (one study) [25] with relevant environmental changes in order to increase children’s water consumption and decrease their sugar-sweetened beverage consumption (Table 3).

These programs exhibited a good potential for public health impact as they documented improvements in dietary behaviors, such as increased water consumption, decreased free sugar intake and improvement in nutrition-related knowledge. Three interventions were based on the health promotion model [23,24,25], and one of them was also based on the ecological model [24]. In addition, mixed modes of intervention delivery were found: one study involved teachers’ training and participation of children’s parents [23], another study involved teachers’ training [25], and in the third intervention, only children’s parents participated and there was no teachers’ training [24] (Table 1).

The DIATROFI program was conducted by directly comparing two different intervention approaches, namely food-voucher approach or free daily meal distribution, and found that the meal distribution intervention was considered more effective than the food voucher one, not only because of its pedagogical benefits, but most importantly because it appeared to improve dietary habits, alleviate food insecurity and break stereotypes for parents and children through universal student participation [26] (Table 3). In the DIATROFI intervention, children’s parents participated without teachers’ training (Table 1).

Four unsuccessful diet-only interventions were identified (Table 2 and Table 4). All of them were developed without using a specific theoretical framework. One study involved teacher’s training and parents’ involvement [27]. Three of the unsuccessful studies had a duration of 6 months [27,28,29], while 1 lasted for only 3 days [30]. Even though the three studies reported food knowledge improvement [28,29,30], these results were not translated into lifestyle changes in 2 studies [28,30], whereas in the fourth study, both positive and negative dietary changes were observed (higher consumption of both healthy and unhealthy foods) [29].

### 3.3. Diet and Physical Activity Interventions

Among the successful diet and physical activity interventions, three involved teachers’ training and participation of children’s parents [31,32,33,34], one involved teachers’ training [35] and one involved participation of children’s parents [36]. Teachers’ training was conducted through workshops concerning healthy habits for school children and/or through teaching materials (re-printed posters for drinking rules or pre-prints to record children’s fluid intake).

In one program, teachers delivered specific education contents (overweight and obesity prevention; concepts of food and nutrition, and dietary guidelines for children and families; hydration and the importance of water; strategies to encourage fruit and vegetable consumption and to reduce intake of low-nutrition, energy-dense foods; appropriate physical activity levels and strategies to reduce screen time; healthy cooking activities), and then they further developed creative and engaging classroom activities on the addressed topics. Themed games and modified sports were performed under the following thematic areas: fun, inclusion and cooperation, and safety.

The intervention program of one study was based on the health promotion model and the social cognitive theory [35], and the intervention program of another one was based only on the social cognitive theory [33]. Both interventions improved children’s dietary behavior, and the intervention that lasted for 2 years had beneficial effects on anthropometric measurements and biomarkers, even though this result concerned children of a specific age (10 years of age).

Three studies had a benefit on anthropometric measurements [31,32,33,36]. Specifically, one study showed a reduction in the incidence of obesity [31,32], another one showed an increase in the % of children of healthy body weight [33], and one found a reduction in the waist-to-height ratio, but this reduction was recorded only in 10-year-old children [36]. Furthermore, two studies had a beneficial effect on biomarkers and blood pressure (BP) [33,36]. Specifically, blood glucose and triacylglyceride (TAG) concentrations were reduced following the intervention [33]. Five studies found favorable change for at least one dietary behavior outcome [33,35,36,37,38,39]. These behaviors included regular breakfast consumption [33,37], adequate vegetable consumption, moderation in sodium intake [35] and adequate fiber intake (shown only in 6-year-old boys) [36], and better diet quality [33,39]. When it came to physical activity behavior, one study showed positive effects on physical fitness and motor skills (speed, coordination, strength, mobility and endurance) [34], and another one found decreased time spent on sedentary activities [38,39]. Finally, one study showed improvement in the self-efficacy of children [37].

**Table 1:** General characteristics of successful interventions.

Program Name Reference	Country	Study Design	Duration	Population Group	Parents’ Involvement	Teachers’ Training	Theoretical Model		
							Social Cognitive Theory	Ecological Model	Health Promotion Model
***Diet only***									
*Diatrofi* Dalma et al., 2018 [26]	*Greece*	RCT	1 school year	N = 6288, elementary and secondary schools	X				
*H2NOE Water Schools* Griebler et al., 2021 [23]	*Austria*	non-randomized controlled cluster trial	1 school year	N = 1148, 8 years old	X	X			X
*HKCC* Irwin et al., 2019 [24]	*United Kingdom*	non-randomized CT	1 school year	N = 931, 8–14 years old	X			X	X
*ACTION* Winzer et al., 2021 [25]	*Austria*	RCT	5 weeks	N = 344, 5th grade		X			X
***Diet and PA***									
*POIBA* Ariza et al., 2019 [31]	*Spain*	multicomponent and multilevel RCT	1 school year	N = 3073, 9–10 years old	X	X			
*POIBA* Sánchez-Martínez et al., 2021 [32]				N = 1653, 9–10 years old	X	X			
- Altay et al., 2020 [37]	*Turkey*	RCT	6 weeks	N = 1609, 9–15 years old					
*Health Promotion Intervention to Improve Diet Quality in Children* Rosário et al., 2017 [35]	*Portugal*	RCT	6 months of teachers’ training/5-month implementation	N = 294, 6–12 years old		X	X		X
*SMS* Weber et al., 2017 [34]	*Germany*	non-randomized CT	10 months	N = 192, 8–10 years old	X	X			
*HHP* Pablos et al., 2018 [33]	*Spain*	RCT	8 months	N = 158, 5th or 6th grade	X	X			
*Project Spraoi* Merrotsy et al., 2019 [36]	*Ireland*	RCT	2 years	N = 101, 6 and 10 years old	X				X
*HPSF* Bartelink et al., 2019 [38,39]	*The Netherlands*	non-randomized CT	2 years	N = 1974 4–12 years					

**Table 2:** General characteristics of unsuccessful interventions.

Program Name Reference	Country	Study Design	Duration	Population Group	Parents’ Involvement	Teachers’ Training	Theoretical Model		
							Social Cognitive Theory	Ecological Model	Health Promotion Model
***Diet only***									
- Verdonschot et al., 2020 [28]	*The Netherlands*	non-randomized CT	6 months (follow-up: 6 months)	N = 1274, 7–12 years old	Χ				
*Project Daire* Brennan et al., 2021 [29]	*Ireland*	RCT	6 months	N = 903, 6–7 and 10–11 years old					
*Taste Lessons* Battjes-Fries MC et al., 2016 [27]	*The Netherlands*	non-randomized CT	6 months follow-up of 1mo and 6 months	N = 392, 8–12 years old		X			
- Weber et al., 2020 [30]	*Germany*	non-randomized CT	3 days (follow-up: 3 months)	N = 305, 8–10 years old					
***Diet and PA***									
*Extra Fit* Kocken et al., 2016 [40]	*Thw Netherlands*	RCT	2 school years	N = 1112, 9–11 years old	X				
*KOP* Mack et al., 2020 [41]	*Germany*	RCT	2 weeks (follow-up: 4 weeks)	N = 82 9–12 years					

**Table 3:** Components of successful intervention programs.

Program Name Reference	Intervention	Outcome Measures		
		Anthropometrics	Nutrition Behavior	Other
***Diet only***				
*Diatrofi* Dalma et al., 2018 [26]	Intervention A: daily lunch bag Intervention B: food voucher		↑ positive food preferences at school	↑ self-organization
*H2NOE Water Schools* Griebler et al., 2021 [23]	Free refillable water bottle and workshops		↑ water consumption	
*HKCC* Irwin et al., 2019 [24]	Education programs about water and SSB consumption and water bottle filling stations		↑ water consumption ↓ SSB consumption	↑ nutrition knowledge
*ACTION* Winzer et al., 2021 [25]	Diet and hydration sessions		↓ free sugar intake	
***Diet and PA***				
*POIBA* Ariza et al., 2019 [31]	Evaluation of body weight, diet and PA sessions Family workshops Subsidized fees for extracurricular sports for some families	↓ Incidence of obesity		
*POIBA* Sánchez-Martínez et al., 2021 [32]		↓ Incidence of obesity		
- Altay et al., 2020 [37]	Evaluation of body weight, healthy lifestyle sessions, workshops and booklets		↑ breakfast consumption	↑ self-efficacy
*Health Promotion Intervention to Improve Diet Quality in Children* Rosário et al., 2017 [35]	Interactive overweight and obesity prevention, diet, PA, hydration and cooking sessions Sessions delivered by trained teachers who took the same sessions		↑ vegetable adequacy ↑ sodium moderation	
*SMS* Weber et al., 2017 [34]	2 additional exercise lessons weekly from qualified trainers and 10 nutrition lessons per school year	NS BMI, muscle and fat mass, and percentage body fat	NS changes in self-reported food consumption	↑ physical fitness and motor skills
*HHP* Pablos et al., 2018 [33]	PA sessions and activities Interactive healthy habits sessions Sessions delivered by trained teachers Worksheet completion about healthy habits Workshops for families and teachers	↑ prevalence for BMI according to level	↑ proper breakfast consumption ↑ better diet quality	↓ glucose ↓ TAG ↓ BP ↑ VO_2max_
*Project Spraoi* Merrotsy et al., 2019 [36]	PA and nutrition sessions and PA classes	↓ waist-to-height ratio (only 10-year-old children)	↑ fiber intake (only 6-year-old boys)	↓ systolic and diastolic BP (only 10-year-old children)
*HPSF* Bartelink et al., 2019 [38,39]	Full HPSF: free mid-morning snack and lunch every day, structured PA sessions and cultural activities, water bottles Partial HPSF: structured PA sessions and cultural activities	Full HPSF ↑ healthy dietary behaviors ↑ school water consumption Partial HPSF ↓ unhealthy dietary behaviors		Full HPSF ↓ time spent sedentary ↑ time in light PA ↓ total time spent in both PA and sedentary behaviors
			At school ↑ water consumption (full HPSF)	At school ↓ time spent sedentary ↑ time in light PA and MVPA increased (full and partial HPSF) At home ↓ time spent in light PA (partial HPSF)

BMI: body mass index, BP: blood pressure, MVPA: moderate-to-vigorous PA, NS: non-significant, PA: physical activity, SSBs: sugar-sweetened beverages, TAG: triacylglycerides, VO_2max_: maximal oxygen uptake.

**Table 4:** Components of unsuccessful intervention programs.

Program Name Reference	Intervention	Outcome Measures		
		Anthropometrics	Nutrition Behavior	Other
***Diet only***				
- Verdonschot et al., 2020 [28]	FV: 3 pieces of FV per child per week Ed group: 5 nutrition lessons FV + Ed group		NS change in consumption of FV	↑ nutrition knowledge
*Project Daire* Brennan et al., 2021 [29]	Nourish: provision of healthy snacks, resources to improve school food presentation, cookery equipment and recipes, sensory education materials, catering for school events, attendance at Tasting Days, holding discussions with relevant school staff Engage: lessons + activities developed to support the lesson. The intervention was largely delivered by teachers		Nourish intervention ↑ positive changes ↑ fizzy drinks and chocolate	Nourish Intervention ↑ emotional and behavioral wellbeing ↑ food knowledge ↑ cooking competence
*Taste Lessons* Battjes-Fries MC et al., 2016 [27]	10–12 lessons per two grades (grades 5–8). Each lesson included taste testing, conducting experiments and homework assignments.			Teachers and children highly appreciated the taste lessons NS change in eating behavior
- Weber et al., 2020 [30]	3 practical nutrition lessons from dietitians			↑ nutrition knowledge ↑ nutrition skills (prepared fruit quark by themselves) NS change in behavioral development
***Diet and PA***				
*Extra Fit* Kocken et al., 2016 [40]	7 nutrition and physical activity lessons in the 1st school year, and 9 lessons in the 2nd year	NS changes in BMI, waist and hip circumference	NS changes in consumption of breakfast and specific food groups (fruits, vegetables, sweet drinks, savory and sweet snacks)	↑ nutrition knowledge
*KOP* Mack et al., 2020 [41]	Playing a game twice a week (45 min): 1. presentation of a story in a video; 2. the player has to move an avatar in a 3D medieval world to walk from the site of one task to another Topics addressed by the game: nutrition, physical activity and stress coping		NS changes in HNI	↑ food knowledge NS changes in PA

BMI: body mass index, FV: fruits and vegetables, HNI: Healthy Nutrition Index, NS: non-significant, PA: physical activity.

We detected two unsuccessful diet and physical activity interventions [40,41]. None of these interventions were based on a theoretical model and there was no teachers’ training in their study protocol. One intervention included children’s parents in the design [40]. Both of them showed significant positive impact on nutrition knowledge. However no major effects were found for their primary outcomes (anthropometric, dietary and physical activity determinants) [40,41].

## 4. Discussion

The present review aimed to summarize available evidence regarding school-based interventions for promoting healthy lifestyle behaviors in children in Europe, as well as potentially identify common characteristics in the successful ones in comparison with those that did not produce the expected results in lifestyle behaviors. The review specifically focused on the findings of interventions that aimed to impact on any kind of health outcomes. We observed that successful interventions on diet and physical activity had favorable effects on a series of health outcomes, such as anthropometric measurements, biomarkers, eating behavior and self-efficacy. As expected, effective diet-only interventions had a positive impact only on specific eating behaviors. It was also noticed that the majority of successful interventions included the participation of children’s parents and/or teachers’ training and lasted for 1 school year, while half of the included studies were based on a theoretical model. Interestingly, all effective school-based interventions, except for one [26], included in their design at least two of the following components: teachers’ training, parental involvement and a theoretical model. On the contrary, unsuccessful school-based interventions were not based on a well-defined theoretical framework. Furthermore, all of them included none or only one of the aforementioned components, and five of the six studies lasted less than one year [27,28,29,30,41].

Some successful school-based interventions described in the current review had an impact on more outcomes than others. These interventions aimed to promote both nutrition and physical activity, and they produced improvements in a series of health outcomes [33,36]. The results of previous works that included studies outside of Europe are consistent with our findings. A review conducted in 2014 suggested that interventions promoting healthy eating habits and physical activity at school should be a strategy in the prevention of obesity [42]. The aforementioned review included interventions delivered in primary schools (children aged up to 12 years) and examined the impact of these interventions on BMI or any anthropometric measures (weight, waist circumference or other indicators of adiposity), and less than half of the studies were performed in Europe.

Regarding the mode of delivery, it is widely accepted that parents play a central role in shaping children’s eating patterns and, consequently, it is advocated that interventions, aiming at improving children’s lifestyle behaviors, need to address the family [43,44]. In the studies included in the present review, children’s parents were involved in different ways: they were recipients of information material about healthy food habits (lunchbox ideas, hydration, tasting sessions and education sessions on high-sugar beverages and takeaways), they participated in family workshops on food and physical activity at school, they supported their children in their tasks for the program (assisting children’s self-monitoring regarding eating and drinking habits), and they were beneficiaries of food vouchers. Moreover, parents participated in focus groups about perceptions regarding the program, triggers and barriers of participation, attitudes toward the program and suggestions for improving the program’s implementation. Two previous reviews showed that school-based interventions with parental involvement had the potential to improve children’s weight status, physical activity and sedentary behavior [45,46]. On the other hand, according to the results of a systematic review conducted in 2012 on combined community- or school- and home-based obesity prevention interventions, only 7 out of the 15 studies produced beneficial changes in lifestyle outcomes, namely eating behavior, physical activity levels, sedentary behavior, or weight status and other health risk factors [47]. It should be added that this review did not focus on school-based interventions as it included community-based ones too. Another possible explanation for the inconsistencies between our findings and those of the 2012 review is the different age groups investigated. We examined interventions performed in primary school children, while the previous review included interventions performed in children aged from 1 to 18 years. We observed that parental involvement was a common component among effective school-based interventions, while the majority of unsuccessful ones lacked this characteristic.

It has been reported that teachers’ training is an effective strategy at improving physical activity of students [48]. This is consistent with the findings of the present review as we observed that training of teachers is a practice often applied by effective school-based interventions and, at the same time, missing from the unsuccessful ones. Therefore, parental involvement and teachers’ training were found to be the most common components of successful school-based interventions.

Previous reviews had pointed out the heterogeneity of the studies they included in terms of design. We noticed that the majority of successful interventions had a duration of 1 school year, whereas the majority of unsuccessful ones lasted for 6 months [27,28,29,30,41]

Additionally, half of the successful studies used a theoretical model for the design of the intervention, whereas ineffective school-based interventions were not based on a well-defined theoretical framework. This is consistent with a previous review conducted in 2006, addressing the important role of the social cognitive theory when designing interventions for preventing and treating childhood obesity. Specifically, this review aimed to describe theoretical and methodological characteristics of effective school-based interventions resulting in a significant decrease in BMI or weight in children aged 4 to 14 years (2/10 studies were European). We found that the inclusion of a theoretical model in the intervention programs led to the improvement of other parameters, mostly in dietary behavior [49].

In this work, we focused on school-based interventions with the aim of identifying and comparing components of successful and unsuccessful ones. We included only controlled trials, as they are considered to be the gold standard for drawing conclusions [50]. Studies without published outcomes were excluded as it was not possible to evaluate their efficacy. We also evaluated the interventions’ effectiveness over a series of health outcomes, and we did not focus solely on anthropometric and physical activity measures.

The limitations of this work should also be acknowledged. This is not a systematic review. We included only studies conducted in European countries that were published in three databases. While these databases are quite extensive, they do not include studies published in another language other than the English language. Therefore, publication bias cannot be excluded. However, to counterbalance this limitation, the gray literature was also searched. Despite these limitations, the current study provides an up-to-date overview of all strategies whose practices had been used in Europe to promote healthy lifestyle behaviors among children.

## 5. Future Directions and Conclusions

To conclude, interventions combining diet and physical activity had favorable results in a series of health outcomes, such as anthropometric parameters, biomarkers and lifestyle behaviors. It should also be noted that most successful interventions included parental involvement, teachers’ training, and lasted for 1 school year, and half of them were based on a theoretical model. Furthermore, the majority of successful interventions consisted of at least two of the aforementioned components. It seems that there is no one single parameter of success and several approaches and strategies need to be included in an intervention. Some effective strategies were identified, but one may argue that the one-size-fits-all approach may not be applicable to all settings. Future research should focus on improving the quality of the evidence and expanding implementation settings. Consequently, more studies with a rigorous study design are needed, in other words, studies with an appropriate sample size, a relevant control group, an appropriate follow-up period well beyond post-intervention, and the inclusion of validated measures of dietary and physical activity behaviors, measurements of body composition and assessment of engagement and sustainability issues.

## Data Availability

No new data were created or analyzed in this study. Data sharing is not applicable to this article.
